# Endovascular flow reduction after portal vein arterialization: a technical note

**DOI:** 10.1186/s42155-026-00686-8

**Published:** 2026-04-09

**Authors:** Elif Can, Magdalena Menzel, Michael Christian Doppler, Katharina Vogt, Charlotte Wintergerst, Lampros Kousoulas, Sophia Chikhladze, Dietrich Alexander Ruess, Philipp Holzner, Stefan Fichtner-Feigl, Wibke Uller

**Affiliations:** 1 Department of Diagnostic and Interventional Radiology, Medical Center-University of Freiburg, Faculty of Medicine, University of Freiburg, Hugstetter Str. 55, Freiburg, 79106 Germany; 2 Department of General and Visceral Surgery, Center for Surgery, Medical Center-University of Freiburg, Faculty of Medicine, University of Freiburg, Freiburg, Germany

## Abstract

**Purpose:**

Portal vein arterialization (PVA) is a salvage technique used to preserve hepatic and biliary perfusion when hepatic arterial reconstruction is not feasible. Excessive arterioportal inflow, however, may cause clinically relevant portal hyperperfusion with ascites, gastrointestinal or biliary bleeding, and hepatic dysfunction. This technical note describes a practical interventional radiology approach to temporary, graded, and definitive endovascular flow modulation after PVA.

**Materials and methods:**

All consecutive patients with native livers who underwent PVA at a tertiary referral center between February 2020 and February 2024 were retrospectively reviewed. Cases requiring endovascular flow reduction for portal hyperperfusion were analyzed with regard to indication, timing, technique selection, technical feasibility, and short-term clinical course. Technical success was defined as correct device deployment with immediate angiographic reduction of arterioportal inflow. Procedure-related complications were classified according to CIRSE.

**Results:**

Six patients required endovascular flow modulation after PVA, accounting for nine procedures. Balloon occlusion with repositioning was used as a temporary measure and a hemodynamic test when definitive closure was considered premature. Stent-based flow modulation was used for graded reduction when persistent hyperperfusion required decompression but residual shunt perfusion was to be preserved. Coil embolization was used for definitive closure in refractory cases or when complete occlusion was deemed acceptable. Technical success was achieved in 7/9 procedures and clinical success in 5/9. Despite technically successful flow reduction, two patients died from septic multiorgan failure.

**Conclusion:**

Endovascular flow modulation after PVA is technically feasible using complementary IR strategies. Balloon occlusion is useful for temporary or test reduction, stent techniques for partial flow modulation, and coil embolization for definitive closure. Technique selection should be guided by timing after PVA, clinical presentation, and anticipated hepatic tolerance rather than by a uniform treatment algorithm.

## Introduction

In patients with native livers, unreconstructable loss of hepatic arterial inflow may lead to severe biliary ischemia, hepatic dysfunction, and liver failure; PVA is therefore used as a salvage option when direct arterial repair is not feasible [1, 2], as it can maintain hepatic and biliary perfusion. However, diversion of arterial inflow into the portal system can result in clinically significant complications, including portal hypertension with refractory ascites, gastrointestinal or biliary bleeding, hepatic/biliary dysfunction or parenchymal injury, and-in high-flow shunts-systemic hyperdynamic circulation or high-output cardiac strain [3, 4].

Interventional radiology (IR) plays a key role in managing these complications, offering minimally invasive approaches such as coil embolization, balloon occlusion, or stent placement to modulate portal inflow [3, 5–8].

Despite increasing experience with these techniques, evidence regarding the optimal timing, choice of intervention, and durability of endovascular flow reduction after PVA remains limited. In this technical note, we present our single-center experience with IR-based flow modulation after PVA and focus on the practical use of temporary reduction, partial flow reduction, and definitive closure strategies. The aim was to describe the frequency and nature of endovascular procedures performed for portal hyperperfusion, their technical feasibility and short-term clinical course, and the surgical contexts in which endovascular intervention became necessary.

## Technical approach and procedural considerations

### Patient selection and scope

This retrospective technical note included all consecutive patients with native livers who underwent portal vein arterialization (PVA) between February 2020 and February 2024 at a tertiary referral center. Clinical records, imaging studies, and procedural reports were reviewed to identify patients who subsequently underwent interventional radiology (IR) procedures for portal hyperperfusion. Recorded variables included the indication for flow modulation, interval after PVA, access route, devices used, intended hemodynamic effect, and short-term clinical course. No predefined hemodynamic cutoff values or portal flow thresholds were used to trigger intervention; instead, treatment decisions were based on clinical symptoms, cross-sectional imaging, angiographic appearance, and duplex findings when available.

No predefined hemodynamic cutoff values or portal flow thresholds were used to trigger intervention. Decision-making was based on the combination of clinical symptoms, cross-sectional imaging, angiographic appearance, and duplex findings when available.

### Indications for endovascular flow modulation

Endovascular intervention was considered in the presence of clinically significant portal hyperperfusion after PVA. The principal indications were refractory ascites, upper gastrointestinal or biliary bleeding, persistent high-flow arterioportal shunting on imaging, and clinical or radiologic evidence suggesting ongoing portal hypertension despite conservative management. The goal of the intervention was not uniform shunt elimination in all cases, but rather hemodynamic tailoring according to the clinical context: temporary flow reduction, partial flow reduction, or complete occlusion.

### Imaging assessment and technique selection

Technique selection was guided by the clinical presentation, the time interval after PVA, and the angiographic or cross-sectional appearance of the shunt.

Temporary balloon occlusion was selected when portal hyperperfusion required immediate but potentially reversible decompression, particularly when complete shunt closure was considered unsafe because hepatic or biliary perfusion might still depend on the PVA. Balloon occlusion was therefore used both as a therapeutic maneuver and as a functional test of hepatic tolerance to reduced shunt flow.

Stent-based flow modulation was selected when the aim was graded lumen reduction rather than immediate occlusion, particularly in conduit-type shunts or in anatomies where preservation of residual flow was considered desirable. Sandglass-shaped covered stents or stent-in-stent techniques were used to achieve controlled narrowing while maintaining shunt patency.

Coil embolization was selected for definitive occlusion when complete shunt closure was judged acceptable, particularly in refractory bleeding or in cases where persistent hyperperfusion remained clinically relevant despite prior temporary or partial reduction.

### Access routes and technical setup

**Fig. 1:**
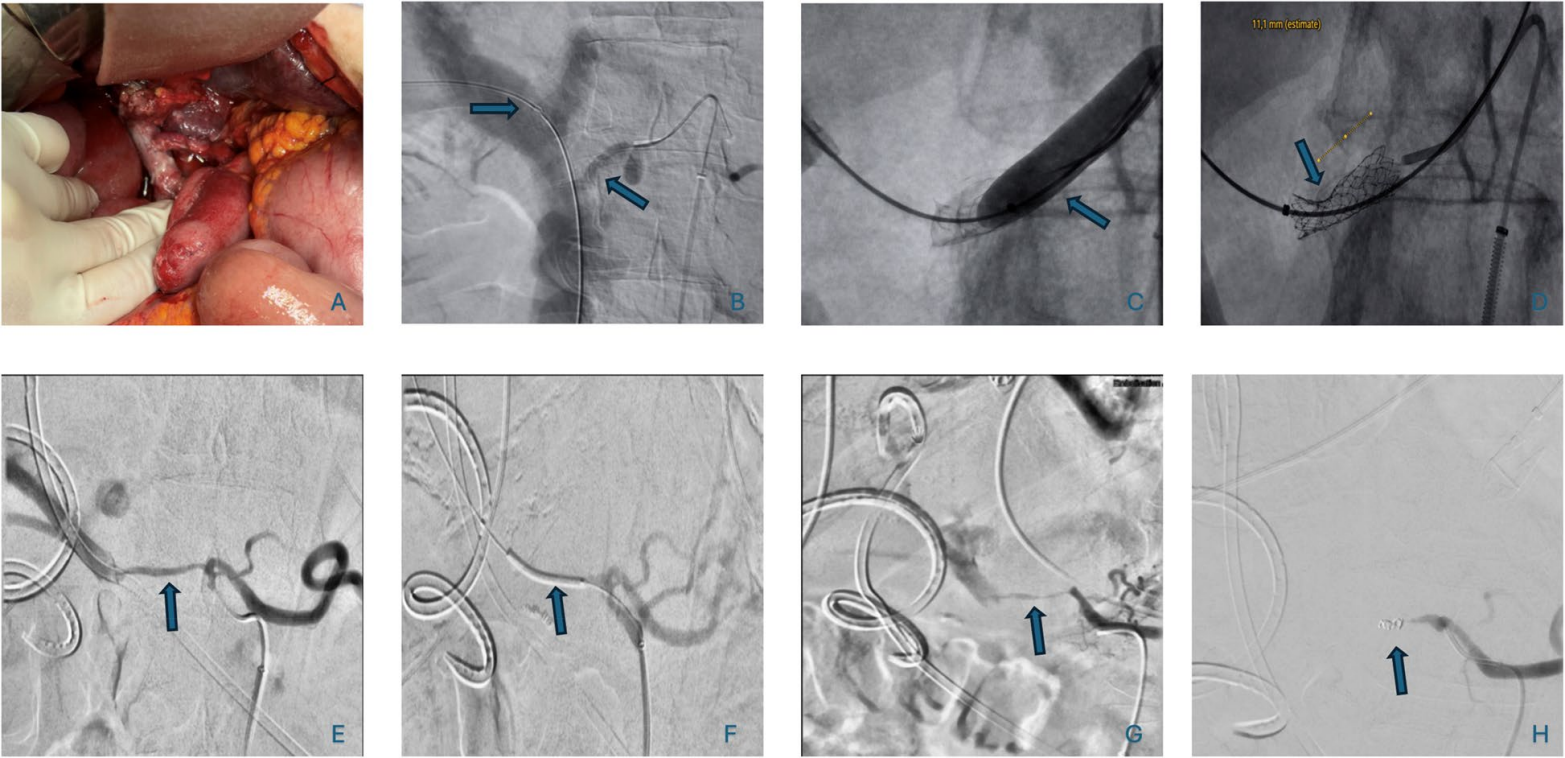
Representative cases of endovascular flow modulation after portal vein arterialization (PVA). A–D Graded stent-graft flow reduction in a 63-year-old woman with pancreatic cancer who underwent a Whipple procedure with surgical PVA; flow modulation was performed 219 days after surgery because of refractory ascites and persistent shunt flow. **A** Intraoperative view of the surgical PVA. **B** Initial angiographic delineation of the arterioportal communication after right transfemoral arterial access; because stable transarterial positioning was limited, additional transhepatic portal access was established. Arrows indicate the transarterial catheter course and the arterioportal shunt/anastomotic region. **C** Balloon-assisted shaping of the shunt segment during graded reduction. A balloon-expandable covered stent graft (BeGraft™, Bentley InnoMed GmbH, Hechingen, Germany; 6×22 mm) was positioned across the communication and molded with balloon assistance into a sandglass configuration. The arrow indicates the shaping balloon. **D** Final angiographic appearance after covered stent-graft implantation with a deliberate central waist for controlled flow reduction while preserving residual perfusion. The arrow indicates the narrowed central segment (sandglass configuration). Additional materials included a 5F arterial sheath, 4F SIM-1 catheter, 4F RIM catheter, 6F transhepatic sheath, a stiff guidewire (Terumo, Tokyo, Japan), and transhepatic tract closure using a Glubran®2/Lipiodol® Ultra Fluid mixture (1:3, v/v; Glubran®2, GEM S.r.l., Viareggio, Italy; Lipiodol® Ultra Fluid, Guerbet, Villepinte, France). **E**–**H** Staged temporary balloon occlusion followed by definitive coil embolization in a 66-year-old patient with pancreatic head carcinoma after pylorus-preserving pancreatoduodenectomy (PPPD) and PVA, first treated 55 days after PVA because of hypertensive gastropathy with recurrent upper gastrointestinal bleeding. **E** Baseline angiography demonstrating the persistent arterioportal fistula/shunt (arrow). **F** Temporary balloon occlusion of the fistula using a low-profile balloon catheter (2×20 mm) after selective catheterization with a 2.4-F PROGREAT™ microcatheter (Terumo, Tokyo, Japan) via right transfemoral arterial access. The arrow indicates the balloon positioned within the fistula. **G** Follow-up intervention with definitive coil embolization after persistent shunt flow despite temporary balloon reduction and repositioning. An AZUR™ coil system (Terumo, Tokyo, Japan; 3 mm/4 cm and 2 mm/2 cm) was selectively deployed into the fistula. The arrow indicates the embolization site. **H** Final angiographic control demonstrating complete occlusion of the arterioportal fistula with preserved perfusion of non-target arterial branches. The arrow indicates the former shunt site after embolization

Access strategy was adapted to shunt anatomy and the intended intervention. Most procedures were initiated through common femoral arterial access using 4F–6F systems for selective celiac, mesenteric, or graft angiography. When stable arterial support was not achievable or when the arterioportal communication was more safely crossed from the portal venous side, transhepatic portal venous access was added under ultrasound guidance. In selected graft-based shunts, combined or bilateral femoral access was used to permit simultaneous device control and lumen shaping. Commonly used macrocatheters included Simmons‑1 (SIM1/“Sidewinder”), Cobra (C2), as well as RIM catheters (AngioDynamics, Latham, NY, USA). Microcatheter support was obtained with PROGREAT® systems (Terumo, Tokyo, Japan). Vascular sheaths (Terumo, Tokyo, Japan) were used for arterial and transhepatic access. Standard and stiff guidewires, as well as 0.014‑inch support wires, were selected according to the need for shunt crossing, stent delivery, or controlled balloon positioning. Covered stents were used for partial flow reduction, detachable coils for complete occlusion, and low-profile balloons for temporary flow reduction or intrastent shaping. When transhepatic access was used, the puncture tract was embolized at the end of the procedure. The nominal stent diameter was selected to match the conduit or landing zone, whereas flow reduction was achieved by deliberate creation of a central waist. Temporary balloon occlusion was used as a staged method of flow reduction rather than as a definitive embolic treatment In selected cases, the balloon catheter was left in place for continued temporary occlusion and was reassessed by repeat fluoroscopy and duplex ultrasound of the liver vessels before repositioning, continuation, or conversion to definitive embolization. This approach was intended to simulate shunt reduction and to assess clinical tolerance before committing to permanent closure. Balloon size was chosen individually according to the angiographic shunt caliber and the intended degree of temporary flow reduction rather than complete exclusion. (Tables 1 and 2, Fig.1)

**Table 1:** Cohort context and patient-level endovascular flow-modulation after portal vein arterialization

Panel A. Clinical context and early mortality in the overall cohort (*n* = 20)											
**Factor**	**Subgroups**	**Patients (n)**	**Early deaths (n)**	**Mortality (%)**							
Age	< 70 vs ≥ 70 years	13 vs 7	4 vs 3	31 vs 43							
Sex	Male vs Female	13 vs 7	4 vs 3	31 vs 43							
Malignancy	Yes vs No	17 vs 3	5 vs 2	29 vs 67							
Complete liver mobilization	Yes vs No	7 vs 13	4 vs 3	57 vs 23							
Major bile duct resection	Yes vs No	14 vs 6	7 vs 0	50 vs 0							
Arterial flow interruption	Complete vs Partial	13 vs 7	5 vs 2	38 vs 29							
Panel B. Patient-level endovascular flow-modulation strategies (6 patients, 9 procedures)											
**Case**	**Interval from surgery/PVA to first IR**	**Clinical indication**	**MBDR**	**IR strategy sequence**	**Access route(s)**	**Key device(s) and size(s)**	**Intended hemodynamic effect**	**Technical success**	**Immediate angiographic endpoint**	**Short-term clinical course**	**Highest CIRSE grade**
1	26 days after right hemihepatectomy	Persistent portal hypertension after PVA via the right hepatic artery arising from the SMA	Yes	Definitive coil embolization	Right femoral arterial	AZUR™ detachable coil (Terumo, Tokyo, Japan), 5 mm/20 cm	Definitive closure	Yes	Occlusion of former arterial inflow to the portal vein with preserved SMA opacification and indirect portal venous filling	Stable	1A
2	21 days after pancreatoduodenectomy	Diagnostic angiography for hepatic artery occlusion; attempted recanalization	No	Diagnostic/bailout angiography with failed recanalization	Right femoral arterial	Microcatheters and guidewires	Diagnostic/bailout	No	No distal recanalization achieved; **intrahepatic extravasation** during probing attempts	No effective flow modulation achieved	3A
3	Not stated in the procedural report	Portal hypertension with hydropic decompensation and persistent bile leak in an iliacoportal prosthetic shunt	Yes	Sandglass stent-graft implantation	Bilateral and additional right femoral arterial access	BeGraft™ (Bentley InnoMed GmbH, Hechingen, Germany), 6 × 28 mm; Coyote™ balloon (Boston Scientific, Marlborough, MA, USA), 1.5 × 40 mm; Sterling™ balloon (Boston Scientific, Marlborough, MA, USA), 2.5 × 60 mm	Graded reduction	Yes	Marked reduction of shunt diameter	Stable	1A
4	219 days after Whipple procedure (first IR); 225 days (repeat IR)	Refractory ascites with persistent shunt flow after PVA	Yes	Transhepatic stent-graft reduction followed by stent-in-stent tightening	Right femoral arterial plus transhepatic portal access; repeat transhepatic portal access	BeGraft™ covered stent graft (Bentley InnoMed GmbH, Hechingen, Germany), 6 × 22 mm; then BeGraft™ coronary (Bentley InnoMed GmbH, Hechingen, Germany), 4 × 12 mm	Graded reduction	Yes	Reduced shunt flow with preserved splenic and left gastric artery perfusion	Persistent symptoms requiring staged reintervention	2*
5	28 days after tumor resection and PTFE bypass creation	Hyperperfusion through PTFE bypass	No	Covered stent-graft narrowing in sandglass configuration	Bilateral plus additional right femoral arterial access	BeGraft™ covered stent graft (Bentley InnoMed GmbH, Hechingen, Germany), 7 × 57 mm; adjunctive balloons: TREK™ (Abbott, Plymouth, MN, USA), 3.5 × 20 mm; EverCross™ (Medtronic, Minneapolis, MN, USA), 6 × 40 mm and 7 × 20 mm	Graded reduction	Yes	Sandglass configuration with reduction of shunt lumen from 7 mm to approximately 3 mm	Stable	2*
6	Not stated in the procedural report	Recurrent upper gastrointestinal bleeding/hypertensive gastropathy after PPPD and PVA	Yes	Temporary balloon occlusion → balloon repositioning → definitive coil embolization	Right femoral arterial	Low-profile balloon (2 × 20 mm; manufacturer not recorded), then AZUR™ detachable coils (Terumo, Tokyo, Japan), 3 mm/4 cm and 2 mm/2 cm	Temporary test reduction followed by definitive closure	Yes	Final complete occlusion of the arterioportal fistula with preserved flow in the truncus coeliacus, left gastric artery, and splenic artery	Shunt occluded	3A*

Early mortality in panel A refers to death within 60 days after portal vein arterialization. Major bile duct resection (MBDR) was defined as extrahepatic bile duct resection requiring biliary reconstruction. Arterial flow interruption refers to complete vs partial loss of native hepatic arterial inflow at the end of the index procedure. In panel B, timing is given where explicitly documented in the procedural report. CIRSE grading in staged cases refers to the highest documented procedure-related grade within the respective case sequence. (*) denotes cases with staged or repeated interventions

*Abbreviations*: *PVA* portal vein arterialization, *IR* interventional radiology, *PPPD* pylorus-preserving pancreatoduodenectomy, *SMA* superior mesenteric artery, *CIRSE* Cardiovascular and Interventional Radiological Society of Europe

**Table 2:** Procedural summary and practical endovascular strategies after portal vein arterialization

Panel A. Summary of endovascular flow-modulation procedures								
**Parameter**	**Value**							
Patients requiring intervention	6/20 (30%)							
Total endovascular procedures	9							
Flow-reduction procedures	8							
Intended strategies	Definitive closure, partial modulation, temporary reduction							
Techniques used	Coil embolization (*n* = 5), balloon occlusion (*n* = 2), stent placement (*n* = 2)							
CIRSE classification	Grade 1 (*n* = 2), Grade 2 (*n* = 4), Grade 3 (*n* = 3)							
Technical success (flow reduction)	7/8 (88%)							
Clinical success (symptom relief without reintervention)	5/9 (56%)							
Major bile duct resection in intervention patients	5/6 (83%)							
In-hospital mortality in patients requiring intervention	2/6 (33%)							
Median interval PVA → first intervention	59 days (range, 10–105 days)							
Main indications treated	Refractory ascites, biliary hemorrhage, upper gastrointestinal bleeding							
Panel B. Practical endovascular strategies used in this series								
**Technique**	**Used in this series for**	**Typical clinical context in this series**	**Typical access route(s)**	**Representative device(s) and size(s) in this series**	**Sizing principle in this series**	**Intended hemodynamic effect**	**Technical endpoint**	**Main limitation/escalation pathway**
Balloon occlusion	Temporary decompression and functional testing before definitive treatment	Acute bleeding or uncertain tolerance to permanent closure	Transfemoral arterial	Low-profile balloon (2 × 20 mm; manufacturer not recorded); Coyote™ balloon (Boston Scientific, Marlborough, MA, USA), 1.5 × 40 mm	Balloon diameter was selected according to the angiographic shunt/fistula caliber to achieve temporary flow reduction rather than permanent exclusion	Temporary reduction	Immediate angiographic decrease in shunt flow	May require repositioning or conversion to coil embolization
Stent-based flow modulation	Persistent hyperperfusion where residual shunt perfusion was intended to be preserved	Conduit-type shunts, persistent ascites, hydropic decompensation, delayed intolerance of shunt flow	Transfemoral arterial alone, combined arterial access, or transhepatic portal access	BeGraft™ (Bentley InnoMed GmbH, Hechingen, Germany), 6 × 28 mm and 6 × 22 mm; BeGraft™ coronary (Bentley InnoMed GmbH, Hechingen, Germany), 4 × 12 mm; BeGraft™ covered stent graft, 7 × 57 mm, with adjunctive balloons: TREK™ (Abbott, Plymouth, MN, USA), 3.5 × 20 mm; Sterling™ (Boston Scientific, Marlborough, MA, USA), 2.5 × 60 mm; EverCross™ (Medtronic, Minneapolis, MN, USA), 6 × 40 mm and 7 × 20 mm	Nominal stent size matched the proximal and distal landing zones or conduit diameter; flow reduction was achieved by creation of a deliberate central waist rather than by undersizing the stent itself	Graded reduction	Reduced shunt caliber and delayed shunt flow with preserved residual perfusion	May require staged tightening, including stent-in-stent implantation
Coil embolization	Definitive closure of persistent shunt flow	Refractory hyperperfusion or bleeding, or after prior temporary reduction	Transfemoral arterial with superselective microcatheterization	AZUR™ detachable coil(s) (Terumo, Tokyo, Japan), 5 mm/20 cm; and 3 mm/4 cm and 2 mm/2 cm	Coil size was selected according to the caliber of the target artery or fistula and the need for stable, dense occlusion	Definitive closure	Complete angiographic shunt occlusion	Irreversible; should be used only when complete closure is considered acceptable

Panel A summarizes all procedures and outcomes descriptively. One diagnostic/bailout angiographic procedure without intended flow reduction was excluded from the calculation of technical success (flow reduction). Mortality data are provided for clinical context only and should not be interpreted as proof of procedure-related causality

Panel B summarizes the practical procedural logic used in this series and is intended as a technical reference rather than a formal treatment algorithm

### Technical endpoints

The primary endpoint was technical success, defined as successful execution of the intended endovascular strategy with immediate angiographic reduction of arterioportal inflow. Because the procedural goal differed between cases, technical success included temporary reduction, partial flow reduction, or complete occlusion when this represented the intended treatment effect. Clinical success was defined as short-term improvement of hyperperfusion-related symptoms, such as reduction of ascites or cessation of gastrointestinal or biliary bleeding, without immediate need for repeat intervention. Mortality was recorded descriptively as early in-hospital death (≤ 60 days after PVA) or late death (> 60 days after PVA). Procedure-related adverse events were classified according to the CIRSE classification system [9].

### Statistical analysis

Data were analyzed descriptively. Continuous variables are reported as median and range, and categorical variables as absolute numbers and percentages. No formal hypothesis-testing framework was applied, as the purpose of this technical note was to describe procedural indications, technique selection, and short-term technical and clinical outcomes rather than to perform comparative outcome modeling.

## Results

### Patient characteristics

Between February 2020 and February 2024, a total of 20 patients underwent portal vein arterialization (PVA). The decision to create PVA was made intraoperatively when arterial reconstruction was not feasible or when arterial sacrifice was required for oncologic clearance. In our institution, these decisions were made by the attending hepatopancreatobiliary surgeon within the operative team, according to the intraoperative anatomy and reconstructive options available. PVA was not limited to a single surgical operator; however, all cases followed the same institutional principle of using PVA only when arterial continuity could not be restored adequately. Thirteen patients were male (65%) and seven female (35%), with a median age of 60.5 years (range 5-83 years). The majority of patients (85%) were treated for malignant disease, most commonly cholangiocarcinoma (*n*=9) and pancreatic adenocarcinoma (*n*=4). Other malignant entities included hepatocellular carcinoma (*n*=2), colorectal liver metastases (*n*=2), and papillary bile duct carcinoma *(n*=1). Benign indications were present in three patients (15%), including chronic pancreatitis (*n*=2) and visceral artery aneurysm (*n*=1). One pediatric patient with neuroblastoma has been reported separately [8].

These data describe the clinical background of the cohort and serve to contextualize the interventional management strategies outlined below.

### Clinical course and overall context

Early postoperative mortality in the overall cohort was 35% (7/20 patients). At the last available follow-up, 10 of 20 patients (50%) were alive. Among the 10 deaths, seven occurred during the early postoperative period, while three represented late mortality related to tumor progression (*n*=2) or sepsis with multiorgan failure (*n*=1). Early mortality in the overall cohort reflected the complexity of the underlying salvage setting. Among patients undergoing endovascular intervention, deaths occurred in the context of severe postoperative sepsis and multiorgan failure; a direct causal relationship to the IR procedure itself could not be established.

Patients who subsequently required endovascular flow-reduction procedures frequently had complex surgical courses, often involving extensive hepatopancreatobiliary resections. These observations are reported to provide clinical context for the interventional strategies described and are not intended as a prognostic or comparative outcome analysis (Table 1).

### PVA Techniques and imaging assessment

Portal vein arterialization was performed using three surgical techniques: direct hepatic artery–portal vein anastomosis in 10 patients (50%), splenic artery–portal or splenic vein anastomosis in six patients (30%), and jump grafts from the iliac artery in four patients (20%). In half of the cases (10/20), PVA was created during the index operation, while in the remaining cases it was established during urgent reoperation.

Follow-up imaging (US as first-line modality and contrast-enhanced CT when clinically indicated) was available for all patients at a median of 3 days (range, 0–14 days) after PVA. Imaging was reviewed to assess shunt anatomy and patency and to support clinical and interdisciplinary decision-making regarding the need and timing of endovascular flow modulation.

### Endovascular flow-reduction interventions

Portal hyperperfusion-related complications, including refractory ascites, gastrointestinal bleeding, and biliary hemorrhage, were observed in a subset of patients and prompted referral for interventional radiology-based flow reduction. Early interventions were mainly prompted by acute bleeding or severe early hyperperfusion, whereas later interventions were more often performed for persistent or progressive ascites, sustained shunt flow on follow-up imaging, or delayed clinical intolerance of the arterioportal shunt.

Six of the 20 patients (30%) developed clinically relevant portal hyperperfusion after PVA, presenting with refractory ascites (*n* = 4), upper gastrointestinal bleeding (*n* = 1), or biliary hemorrhage (*n* = 1), and subsequently underwent endovascular intervention. A total of nine endovascular flow-reduction procedures were performed (Tables 1 and 2). The median interval between PVA and the first endovascular flow-reduction intervention was 59 days (range, 10–105 days) (Fig. 1).

Interventional strategies included balloon occlusion (*n* = 2), stent-based flow modulation (*n* = 2), and coil embolization (*n* = 5). Selection of the endovascular technique was guided by timing after PVA, shunt anatomy, and anticipated hepatic tolerance to reduced inflow.

Balloon occlusion was preferentially applied in the early postoperative phase as a temporary and reversible measure, allowing short-term flow reduction and functional assessment of hepatic tolerance. Stent-based techniques were used to achieve controlled partial flow modulation when definitive shunt occlusion was considered unsafe. Coil embolization was reserved for definitive shunt closure, typically at later stages when collateralization or compensatory inflow had developed and persistent hyperperfusion-related complications necessitated complete occlusion.

### Interventional endpoints and technical outcome

The primary interventional endpoint was technical success, defined as correct device deployment with immediate angiographic reduction of arterioportal inflow. Technical success was achieved in 7 of 8 intended flow-reduction procedures (88%). One bailout angiographic procedure without intended flow reduction was excluded from the technical success calculation. Two patients experienced persistent or recurrent hyperperfusion-related complications despite technically feasible interventions and died during the early postoperative course due to systemic complications, including septic shock and multiorgan failure.

### IR-related complications

According to the CIRSE classification system, minor complications (CIRSE grade 1–2) occurred in 6 of 9 endovascular procedures (67%), while major complications (CIRSE grade 3) were observed in 3 of 9 procedures (33%). Minor complications included transient access- or procedure-related events without lasting clinical consequences. Major complications were related to persistent hyperperfusion or bleeding requiring additional management. No CIRSE grade 4–6 complications and no unexpected device-related adverse events were observed (Table 2).

## Discussion

In this series, endovascular flow modulation was used in a subset of patients who developed clinically relevant portal hyperperfusion after PVA. From an interventional perspective, the main practical issue was not whether flow reduction should be performed in general, but when to use temporary reduction, when to apply graded narrowing, and when definitive shunt closure appeared acceptable [3–5, 10]. The technique selected depended primarily on the timing after PVA, the clinical presentation, and the presumed degree of ongoing dependence of the liver and biliary system on arterioportal perfusion. The variation in timing most likely reflects differences in clinical presentation rather than a single uniform pathophysiologic mechanism, with acute hemorrhagic or high-flow presentations occurring early and more gradual portal-hypertensive symptoms presenting later. Balloon occlusion, including balloon repositioning, was used as a temporary and potentially reversible measure when complete occlusion was considered premature. In our practice, this approach served both as immediate decompression and as a functional test of whether reduced shunt flow was clinically tolerated. Stent-based techniques, including funneling and stent-in-stent implantation, were used when the goal was graded flow reduction rather than complete closure [3, 5, 10]. These approaches were particularly useful when persistent hyperperfusion required modulation, but abrupt shunt elimination was considered potentially unsafe because the intervention was performed relatively early after PVA or because the angiographic situation suggested continued dependence on the shunt. In contrast, coil embolization [6, 11] was reserved for definitive closure, mainly in refractory bleeding or persistent hyperperfusion when complete occlusion was judged acceptable on the basis of timing, anatomy, and the presumed development of compensatory perfusion. This stepwise distinction between temporary, partial, and definitive approaches is, in our view, the main technical message of this report [5, 10].

Several technical points deserve emphasis. First, balloon occlusion should be understood as a staged maneuver rather than a definitive treatment, and prolonged or repeated balloon-based reduction may be necessary before conversion to embolization. Second, stent-based narrowing requires individualized sizing and shaping, as the intended effect is flow reduction rather than exclusion. Third, access planning is critical: while transfemoral arterial access was sufficient in some cases, combined or transhepatic portal access was required in others to achieve stable device control. The series also illustrates that procedural success does not necessarily translate into favorable overall outcome, as the postoperative course remained strongly influenced by the underlying surgical and septic context. For that reason, any association between surgical variables and mortality should be interpreted cautiously and not as a direct consequence of the endovascular strategy itself [3, 8]. A further finding was the association of major bile-duct resection with higher early mortality, consistent with prior studies linking extensive biliary surgery to ischemia and cholangitis [1, 3, 4]. However, the suboptimal outcomes may be attributed to multiple contributing factors.

This technical note has several limitations. It is retrospective, includes a small number of intervention cases, and reflects heterogeneous surgical anatomies and clinical scenarios. In addition, no standardized hemodynamic thresholds were available to guide escalation from temporary reduction to graded narrowing or definitive closure. Accordingly, the present work should be read as an experience-based technical description rather than as an outcome study or treatment algorithm. Nevertheless, it provides practical information on how different endovascular techniques can be used sequentially and selectively after PVA, and where their main technical advantages and limitations lie.

Further prospective studies are required to define optimal timing, technique selection, and durability of endovascular flow-reduction strategies after portal vein arterialization, and to determine whether pre-emptive modulation in asymptomatic patients can prevent long-term sequelae such as hepatic/biliary injury or systemic hyperdynamic complications.

## Conclusion

Endovascular flow modulation after PVA can be performed using complementary rather than competing techniques. Balloon occlusion is useful for temporary or test reduction, stent-based techniques for graded flow modulation, and coil embolization for definitive shunt closure. Technique selection should be guided by timing after PVA, clinical presentation, and the presumed need to preserve residual shunt-dependent perfusion.

Take-home message: in patients with post-PVA hyperperfusion, the key interventional decision is not simply whether to close the shunt, but whether the clinical situation calls for temporary reduction, graded narrowing, or definitive occlusion.

## Data Availability

The datasets generated and/or analyzed during the current study are not publicly available due to the retrospective nature of the study and the inclusion of sensitive patient data but are available from the corresponding author on reasonable request.
